# New drug interactions in HIV and HCV

**DOI:** 10.1186/1742-4690-9-S1-I8

**Published:** 2012-05-25

**Authors:** David Back

**Affiliations:** 1 University of Liverpool, UK

## 

Drug-drug interactions (DDIs) remain one of the challenges faced by health care professionals involved in patient management and by researchers seeking to understand the many different mechanisms that may be involved. While in HIV treatment there has been an understanding that checking for DDIs is part of routine management, the development of directly acting antiviral (DAA) drugs is changing the whole approach to the treatment of HCV. DDIs have not really been high on the agenda while pegylated interferon and ribavirin have been the standard of care for Hepatitis C; however the first generation HCV protease inhibitors while representing a huge advance, also present new treatment challenge of awareness and management of DDIs.

In this presentation, some of the key areas of DDIs with a) HIV drugs, b) HCV drugs and c) co infected patients receiving both HIV and HCV drugs will be discussed. While it has long been established that boosted HIV protease inhibitors are likely to interact with other medications metabolised by CYP3A4 (and other enzymes/transporters) to increase drug exposure and that drugs like efavirenz, nevirapine and etravirine are inducers of drug metabolism some of the current challenges are to determine i) the likelihood of interactions where there are no study data to guide us and ii) what happens when a patient switches therapy off an enzyme inducing drug. In relation to the new DAAs, Boceprevir is primarily metabolised by the enzyme aldo-ketoreductase (AKR) but also undergoes metabolism by CYP3A4; telaprevir is metabolised by CYP3A4. In addition, both boceprevir and telaprevir are strong inhibitors of CYP3A4 and are substrates for P-glycoprotein. Co administration of drugs that are highly dependent on CYP3A4 for clearance and for which elevated plasma concentrations are associated with serious or life threatening events means a contraindication with boceprevir and/or telaprevir. However, many other interactions require close monitoring, alteration of drug dosage or timing of administration and here we clearly need help to understand the potential magnitude of an interaction and strategies for patient management. Since targeted drug interaction studies are done in the development programme and then post-licensing there are a limited number of drugs for which there are actual study data. Where there are no data, guidance can only be given based on knowledge of the pharmacology of the respective drugs. This is the approach being used in the resource http://www.hep-druginteractions.org.

One particular area of concern is HIV positive patients who are receiving antiretrovirals (ARVs) since they are relatively highly represented among patients with HCV infection, and are at a high risk of DDIs. Recent drug interaction data presented on telaprevir and boceprevir have reinforced the initial suspicions that we are faced with a whole new challenge of managing multiple interactions in co-infected patients.

Management of DDIs with these exciting new agents certainly poses a challenge and awareness of the potential for DDIs is fundamental for safe prescribing. Think DDIs for the DAAs. But we are still on a steep learning curve and there are unanswered questions.

